# Stepovers and Signal Detection: Response Sensitivity and Bias in the Differentiation of Genuine and Deceptive Football Actions

**DOI:** 10.3389/fpsyg.2018.02043

**Published:** 2018-10-29

**Authors:** Robin C. Jackson, Hayley Barton, Kelly J. Ashford, Bruce Abernethy

**Affiliations:** ^1^School of Sport, Exercise and Health Sciences, Loughborough University, Loughborough, United Kingdom; ^2^Centre for Sports Medicine and Human Performance, Brunel University London, Uxbridge, United Kingdom; ^3^Cardiff School of Sport, Cardiff Metropolitan University, Cardiff, United Kingdom; ^4^Faculty of Health and Behavioural Sciences, The University of Queensland, Brisbane, QLD, Australia

**Keywords:** anticipation, deception, signal detection, perception, bias

## Abstract

The ability to differentiate genuine and deceptive actions was examined using a combination of spatial and temporal occlusion to examine sensitivity to lower body, upper body, and full body sources of visual information. High-skilled and low-skilled association football players judged whether a player genuinely intended to take the ball to the participant’s left or right or intended to step over the ball then take it in the other direction. Signal detection analysis was used to calculate measures of sensitivity (*d′*) in differentiating genuine and deceptive actions and bias (*c*) toward judging an action to be genuine or deceptive. Analysis revealed that high-skilled players had higher sensitivity than low-skilled players and this was consistent across all spatial occlusion conditions. Low-skilled players were more biased toward judging actions to be genuine. Receiver Operating Characteristic (ROC) curves revealed that accuracy on deceptive trials in the lower body and full body conditions most accurately classified participants as high-skilled or low-skilled. The results highlight the value of using signal detection analysis in studies of deceptive actions. They suggest that information from the lower body or upper body was sufficient for differentiating genuine and deceptive actions and that global information concurrently derived from these sources was not necessary to support the expert advantage.

## Introduction

The ability to judge the intentions of an opponent using advance visual information confers an advantage in many competitive sport encounters (Mann et al., 2007). A potential disadvantage of being highly attuned to early visual information is that it leaves performers vulnerable to deception, resulting in misreading the intentions of an opponent characterized by incorrect or inefficient responses (Jackson et al., 2006). In early research on deception, researchers showed that expert players in the French martial art savate (a form of kick boxing) made more ‘false alarm’ responses to fake attacks (‘feints’) than intermediate and novice players (Ripoll et al., 1995). In light of the very different consequences of failing to respond to a genuine attack and responding to a feint, this may well have reflected a strategic or perceptual bias on the part of experts rather than indicating their greater susceptibility to deception. The weight of evidence now supports a clear advantage for high-skilled over low-skilled performers in using kinematic information to judge deceptive intent. This has been shown in studies of deceptive ‘sidestep’ actions (Jackson et al., 2006; Brault et al., 2012; Mori and Shimada, 2013), football penalty kicks (Smeeton and Williams, 2012), football ‘stepovers’ (Bishop et al., 2013; Wright et al., 2013; Wright and Jackson, 2014), and discriminations between genuine and deceptive actions in volleyball (Güldenpenning et al., 2013), handball (Cañal-Bruland and Schmidt, 2009), and basketball (Sebanz and Shiffrar, 2009).

An important question in the perception of deceptive intent concerns the information sources used by skilled and less-skilled performers to discriminate between genuine and deceptive actions. Researchers have shown that experts use information from distributed sources to anticipate action outcomes (Ward et al., 2002; Huys et al., 2008, 2009; Cañal-Bruland et al., 2011; Diaz et al., 2012; Loffing and Hagemann, 2014). For example, expert badminton players became increasingly accurate at judging the depth of a shot as markers for the racket arm, head plus non-racket arm, and lower body were progressively added to those depicting the racket and shuttle. In contrast, recreational players relied more on the arm and racket and did not improve when lower body information was added (Abernethy et al., 2008). Similarly, Williams et al. (2009) showed that tennis players were less able to differentiate between cross-court and ‘inside-out’ forehand tennis shots when local motion from the two shots was selectively interchanged. For skilled players, judgments were impaired when the manipulation was applied to a number of local sources, namely motion of the arm and racket, shoulders, hips, and legs. By contrast, judgments of less-skilled players were only impaired when motion of the arm/racket region was manipulated. From this evidence some researchers have inferred that experts use ‘global’ processing whereas low-skilled or novice performers are more reliant on ‘local’ processing of specific sources of information (Huys et al., 2008; Williams et al., 2009). Greater sensitivity to distributed sources of information need not imply global processing as different sources may be processed sequentially. For example, consistent with the constraints attunement hypothesis (Vicente and Wang, 1998) applied to dynamic anticipation tasks, Abernethy et al. showed that expert badminton players process information in a proximal-to-distal manner. For trials occluded early in the striking action, expert players predicted shot depth more accurately when only the player’s lower body or head plus non-racket arm was visible than when only the arm (holding the racket) or racket was visible. By contrast, in later-occluded actions predictions were more accurate when viewing the racket arm or racket than when viewing the player’s lower body or head plus non-racket arm. While this shows that skilled performers make better use of early proximal information they are also more attuned to sources close to the end effector that undergo the greatest displacement (Abernethy and Russell, 1987; Ward et al., 2002; Jackson and Mogan, 2007; Abernethy et al., 2008; Huys et al., 2009). In a series of studies of how cricket batters anticipate bowler actions, Müller and colleagues concluded that the expert advantage is primarily driven by pick-up of advance information from upper body sources, notably the bowling hand and arm over the time period in which the underlying kinematics undergo the greatest change (Müller et al., 2006, 2010).

Instructions for executing common deceptive actions such as the football stepover and rugby sidestep refer to movements of the lower and upper body. To perform sidesteps, expert coaches instruct players to “step wide with the outside leg at the same time leaning your body weight directly over the top of that foot… drive off the outside leg back inside” (Biscombe and Drewitt, 1997, p. 36). Similarly, to execute a football stepover players are instructed to “Go across the ball with the outside of the right or left foot, feint with the upper part of the body and cut inside” (Simpson and Hesse, 2013, p. 36). In sidestep actions, Brault et al. (2010) found that differences in lower body movement (outer foot displacement and lower trunk yaw), upper body movement (head yaw; upper trunk yaw; and upper trunk roll), and centre of mass (COM) displacement differentiated genuine from deceptive actions and characterized more and less effective sidesteps. Brault et al. (2012) showed that expert players were more attuned to the ‘honest’ COM displacement signal whereas non-players were more attuned to deceptive signals. To determine COM at any given moment requires knowledge of *both* the lower and upper body so implies that skilled judgments of deceptive intent rely on global processing rather than enhanced local processing of specific sources. If this is the case then the ability to differentiate genuine and deceptive actions should be attenuated when this information is unavailable, for example when only the lower body or upper body is visible.

The aim of the present study is to test whether concurrent use of lower and upper body sources of information is necessary for judging deceptive intent in a common deceptive action: the football stepover. To address this question, high-skilled and recreational football players judged the direction an opponent would take the ball under three levels of spatial occlusion in which (1) the whole player, (2) only the player’s upper body, and (3) only their lower body, were visible. To ensure results could be attributed to player motion, full-video and point-light tests were constructed. Point-light displays present key joint centers against a dark background and were developed by Johansson (1973) as a means of isolating information in biological motion from cues relating to form. They have been successfully applied to studies of anticipation in sport as a simple means of isolating kinematic information as the performer interacts with an object.

A limitation of previous research on deceptive actions is that judgment accuracy has been assessed separately for genuine and deceptive actions. This yields important information regarding response accuracy for each type of trial; however, it is limited in at least two ways. First, it does not directly measure a fundamental goal of the task, which is to determine whether the intent conveyed by an action (e.g., a football player showing intent to take the ball to the right) is genuine (she takes the ball to the right) or deceptive (she steps over the ball then takes the ball to the left). This ability is captured by a measure of sensitivity that is derived from *both* the proportion of correct responses for genuine trials *and* the proportion of ‘correct rejections’ in deceptive trials. A second limitation of analyzing genuine and deceptive trials separately is that differences in accuracy might reflect different biases toward judging an action to be genuine or deceptive. For example, higher-skilled performers might obtain higher accuracy scores than lesser-skilled performers on deceptive actions because they are more biased toward judging actions to be deceptive, perhaps born of greater exposure to deceptive actions in competitive play. Analysis originating in signal detection theory (Green and Swets, 1966) enables us to examine these issues but has very rarely been employed in studies of deceptive actions in sport.

To the best of our knowledge, the only study to date to employ signal detection analysis in judgments of deceptive actions in sport was conducted by Cañal-Bruland and Schmidt (2009), who asked skilled handball goalkeepers, outfield players and novices to judge whether penalty throws were genuine or deceptive. While goalkeepers and outfield players showed the same level of sensitivity in differentiating genuine and deceptive actions, only the goalkeepers were biased in favor of judging penalty throws to be fake (i.e., judging the shooter would not release the ball). The authors suggested this might reflect knowledge of situational probabilities of the respective actions or an assessment that there are greater costs associated with missing a deceptive action. The source of bias can also be perceptual and this was neatly illustrated by Witt et al. (2015) in their model of the effect of tail orientation on judgments of line length using the Müller-Lyer illusion. Likewise, perceptual bias applies to deceptive *actions* such as the football stepover, in which the goal of the actor is to ‘fool’ an observer into judging an action to be genuine when it is in fact deceptive. In these tasks the extent to which participant responses are biased toward judging the action to be genuine are an additional measure of the effectiveness of deception and can be assessed at different time points as the action unfolds.

Another feature of signal detection analysis is that one can quantify the degree to which test results differentiate participants on a binary classifier such as membership of a high-skilled and low-skilled group. To do this, Receiver Operating Characteristic (ROC) curves are plotted that depict the rate of true positive identifications (e.g., membership of the high-skilled group) against the rate of false positives (e.g., membership of the low-skilled group) as one progresses through the list of ranked test scores. The area under the curve (AUC) measures the degree to which the test ‘diagnoses’ group membership. This and associated ROC analyses that compare the rates of true positives and false positives for different decision criteria have been extensively applied in a diverse range of fields including medical diagnosis and eye witness identification (Zweig and Campbell, 1993; Swets, 2014; Wixted and Mickes, 2014).

In the present study, we used response accuracy scores to calculate measures of (perceptual) sensitivity (*d′*) and response bias (*c*). ‘Hits’ were defined as correct responses on genuine trials and ‘false alarms’ were defined as incorrect responses on deceptive trials. In the analysis that follows, a *d′* value of 0 indicates an inability to distinguish between genuine and deceptive actions, which can result from any proportion of ‘hits’ on genuine trials as long as it is matched by the same proportion of ‘false alarms’ on deceptive trials. When the proportion of ‘hits’ is greater than the proportion of ‘false alarms’ this will yield positive values of *d′*; conversely, fewer ‘hits’ on genuine trials than ‘false alarms’ on deceptive trials will result in negative *d′* values. In regard to bias, negative values of *c* reflect a bias toward judging actions to be genuine and positive values of *c* reflect a bias toward judging an action to be deceptive. Last, we conducted ROC analysis to examine which elements of the test best differentiated high-skilled and low-skilled participants.

In regard to the measure of sensitivity (*d′*), we hypothesize that (1) sensitivity will be greater for high-skilled players than low-skilled players, reflecting their greater ability to distinguish between genuine and deceptive actions. Consistent with the global processing hypothesis we further hypothesize that (2) sensitivity, and (3) the difference in sensitivity between high-skilled and low-skilled players, will be greater when the whole body is visible than when the upper and lower body are seen in isolation. In regard to the measure of response bias (*c*), we hypothesize that (4) low-skilled players will have a stronger bias toward judging actions to be genuine than high-skilled players. We further hypothesize that (5) bias will be stronger, and (6) the difference in bias between high-skilled and low-skilled players will be greater, in the whole body condition than in the lower body and upper body conditions. In regard to the ROC analysis, we hypothesize that (7) group membership will be better ‘diagnosed’ by judgment accuracy on deceptive trials than genuine trials, and that (8) the AUC will be greatest for the deceptive trials in the full body condition.

## Materials and Methods

### Participants

Forty-eight female football players (24 high-skilled, *M*_age_ = 21.9 years, *SD* = 4.3; 24 low-skilled, *M*_age_ = 21.6 years, *SD* = 1.4) participated in the experiment. High-skilled participants were competing in the Football Association Women’s Super League at the time of the experiment and had a mean of 12.3 years (*SD* = 3.8) of competitive football experience. Low-skilled participants had a mean of 5.1 years (*SD* = 3.5) of recreational football experience. High-skilled and low-skilled participants were randomly allocated to the ‘full video’ and ‘point-light’ test formats. Power analysis was conducted in G^∗^Power (version 3.1, see Faul et al., 2007). For a medium effect size (*f* = 0.25), alpha set at 0.05, and power set at 0.80, the mixed-factor ANOVA calculation yielded a recommended total sample size of 40 for the interaction between group (four levels) and spatial occlusion (three levels), and of 36 for the interaction between group and time of occlusion (four levels).

### Experiment Design and Test Stimuli

The task was designed to simulate a one-on-one football scenario in which one player runs toward an opposing player before attempting to evade the other player by taking the ball to the left or right, with or without a deceptive action. Two skilled female football players with a mean of 13.5 years of competitive National level playing experience were used to create the test stimuli. The video sequences were filmed using a Canon HD digital video camera (Canon HV40, Toyko, Japan) mounted on a tripod at a height of 1.4 m recording at 25 frames per second. For each video clip, the player ran from a starting position located 11.5 m from the video camera and was instructed to change direction in the region of a marker placed 5.3 m from the point directly beneath the video camera lens. At this point, the player moved toward one of two training cones placed at an angle of 45 degrees to the left and right of the initial approach. In the non-deceptive condition, the player was instructed to change direction to the left or right of the camera, while in the deceptive condition the player was instructed to perform a ‘stepover’ by moving their lead foot in front of and across the ball before taking the ball in the opposite direction. The task for participants was to judge whether the approaching player intended to take the ball to their left or right, which required them to judge whether the initial intention conveyed by a movement to the left or right was genuine or deceptive. Participants were told that there would be an equal number of action outcomes to the left and right and an equal number of genuine and deceptive actions.

To select the highest quality actions for the test, three UEFA ‘B’ License football coaches rated each video clip for speed, straightness of approach, and technical execution. The two highest-rated clips for each player changing direction to the left and right with and without a stepover were included in the final test. This generated 16 unique clips, which were then digitally edited using Pinnacle Studio and Jasc Paint Shop Pro software to create three levels of spatial occlusion and four times of occlusion. For simplicity, the three levels of spatial occlusion refer to the information sources that were visible: (A) full body: original video with no areas removed, (B) lower body: each player’s head, arms, hands and torso above the hips were removed, and (C) upper body: each player’s legs, feet and torso from the hips down were removed.

#### Full-Video Stimuli

**FIGURE 1:**
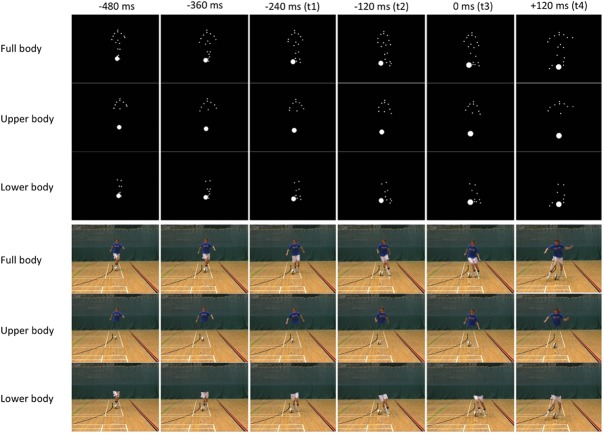
A schematic representation of single frames from the point-light and full video test sequences, showing the three levels of spatial occlusion and four times of occlusion as one of the players performs a stepover. Written informed consent was obtained from the depicted individual for the publication of these images.

To create the spatial occlusion conditions, each frame of the 16 video sequences was edited by cloning a background image of the experiment set up to ‘paint over’ the relevant region of the player. The edited images from consecutive frames were then recombined to create a new video clip. The resulting 48 video stimuli were cropped at four time points relative to the frame before the foot made contact with or passed in front of the ball: t1 (−240 ms), t2 (−120 ms), t3 (0 ms), and t4 (+120 ms) (see Figure 1).

#### Point-Light Video Stimuli

Each frame of the 16 unique video sequences was edited to produce sparse binary (black/white) point-light representations consisting of 19 small disk markers corresponding to principal body joints and extremities (forehead; chin; heads of the left and right humerus; left and right elbow; left and right wrist; navel; left and right iliac spines of the pelvis; left and right patella; left and right heel; mid-points of the lateral and medial malleoli of the left and right ankle; and distal phalanx of the second toe of the left and right foot). In addition, the ball was represented in each frame by a white disk of the same circumference such that the looming effect was retained as the player approached the camera. The 10 markers corresponding to the hips, knees, ankles, and feet of the player were retained to create the ‘lower body’ stimuli. The remaining nine markers were retained to create the ‘upper body’ stimuli (see Figure 1).

The full video and point-light tests each comprised 192 test trials, presented in four blocks of 48 trials. The tests were identical except for display format and were presented on a 15.6” widescreen monitor viewed from a distance of approximately 0.5 m, such that the vertical visual angle subtended by the player at the point of direction change was approximately 10 degrees. In the first two blocks of trials, participants were shown video clips from one of the two players and in the second two blocks were shown video clips from the other player. Player order was counterbalanced across participants to control for possible order effects. The order of trials associated with each player was randomized with respect to levels of deception, spatial occlusion and temporal occlusion. The duration of each trial was approximately 2.0 s and we employed a 5.0 s inter-trial interval.

### Procedure

Institutional ethical approval was granted and all participants gave written consent prior to participating in the study. After completing the participant information and consent forms participants were told that their task was to judge whether the player in the video would take the ball to the left or right of the screen from the participant’s viewing perspective. They were informed that the clips would vary in terms of when they were occluded, that the player would take the ball to the left and right an equal number of times, and that on half of the trials the players would try to deceive them by feigning to take the ball in one direction before moving in the other. Participants were also told the clips would vary in terms of how much of the performer would be visible, namely their whole body, just their upper body, or just their lower body. Participants who viewed the point-light test were informed that the two players would be represented by a group of white dots set against a black background so that sometimes they would see all the dots, sometimes only the dots from the player’s upper body, and sometimes only the dots from the player’s lower body.

Participants were instructed to indicate the direction they thought the player would go by making a verbal response (‘left’ or ‘right’). To familiarize participants with the test format and response requirements, they were shown 16 practice trials in their designated display format (full video or point-light) comprised of eight video clips from each player. These contained examples of each level of deception, spatial occlusion, and temporal occlusion and were generated from different clips to those used in the test.

### Statistical Analysis

The primary dependent variables were sensitivity (*d′*) and bias (*c*), which were calculated for each group in each combination of spatial and temporal occlusion. To calculate *d′*, the proportions of correct responses on genuine trials (‘hits’) and incorrect responses on deceptive trials (‘false alarms’) were converted to *z*-scores. The values for deceptive trials were then subtracted from the values for genuine trials. To calculate *c*, the *z*-scores for deceptive trials were added to those for genuine trials and multiplied by −0.5. To account for the possibility of infinite *z*-scores, values of 0 and 1 were replaced with 1/2n and (n−0.5) ÷ n, respectively, where n is the number of trials in the relevant condition (Stanislaw and Todorov, 1999).

Using these measures and setting up the analysis in this way addresses the key judgment to be made when viewing a step-over action, namely whether the outcome intention initially conveyed by the actor is genuine or deceptive, regardless of whether the intention conveyed is to take the ball to the left or the right. Conceptually, it is important to note that while the participant makes a directional judgment (left or right) rather than one of deceptive intent (genuine or deceptive) the latter is implicit in the former so is subject to analysis for sensitivity and bias. Specifically, a correct response to a genuine action (whether to the left or right) implies a correct judgment that the action was genuine (a ‘hit’). An incorrect response to a deceptive action (whether the initial intention conveyed was to the left or right) implies an incorrect judgment that the action was genuine when it was in fact deceptive (a ‘false alarm’). Conversely, an incorrect response to a genuine action (whether to the left or right) implies an incorrect judgment that the action was deceptive (a ‘miss’). Last, a correct response to a deceptive action (whether the initial intention conveyed was to the left or right) implies a correct judgment that the action was deceptive (a ‘correct rejection’).

A 2 (Expertise: high-skilled, low-skilled) × 2 (Test Display: full video, point-light) × 3 (Spatial Occlusion: full body, upper body, lower body) × 4 (Time of Occlusion: t1, t2, t3, and t4) mixed-factor analysis of variance (ANOVA) was conducted for the *d′* and *c* variables, with expertise and test display entered as between-participant factors, and spatial and temporal occlusion serving as within-participant factors. Alpha was set at 0.05 for all analyses and partial eta squared ($\eta_{p}^{2}$) was used to indicate effect size. The Greenhouse-Geisser adjustment to the degrees of freedom was applied when Mauchly’s test of sphericity was violated. For the ROC analysis, group membership (high-skilled or low-skilled) served as the binary classifier and the AUC was calculated for each combination of spatial and temporal occlusion. In this analysis, classification at chance level produces a diagonal line for the rates of true positives (correct classifications) and false positives (incorrect classifications) so significance is tested against an AUC value of 0.5.

## Results

### Descriptive Data

**FIGURE 2:**
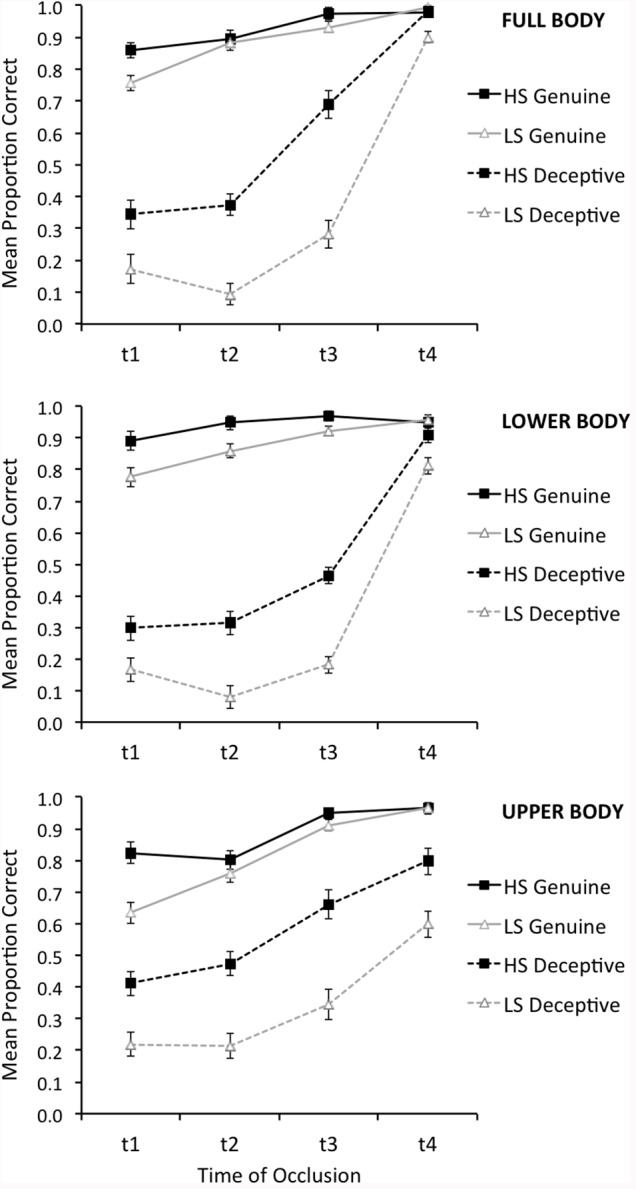
Mean judgment accuracy (±*SE*) for the high-skilled (HS) and low-skilled (LS) participants when judging genuine trials (solid lines) and deceptive trials (dashed lines) at each combination of spatial occlusion and time of occlusion.

The combined accuracy data across the two tests for non-deceptive and deceptive trials in the three spatial occlusion conditions are displayed in Figure 2. In all three spatial occlusion conditions, high-skilled players were slightly more accurate than low-skilled players when judging genuine actions and were considerably more accurate than low-skilled players in judgments of deceptive actions. Although not the primary analysis of interest, this replicates previously reported findings (Brault et al., 2012; Wright and Jackson, 2014) and resulted in a significant Expertise × Deception interaction, *F*(1, 44) = 24.26, *p* < 0.001, $\eta_{p}^{2}$ = 0.36. Overall, there was no significant difference between response accuracy in the full video (*M* = 0.68, *SE* = 0.01) and point-light (*M* = 0.66, *SE* = 0.01) tests, *F*(1, 44) = 3.27, *p* = 0.08, $\eta_{p}^{2}$ = 0.07.

### Signal Detection Analysis

**FIGURE 3:**
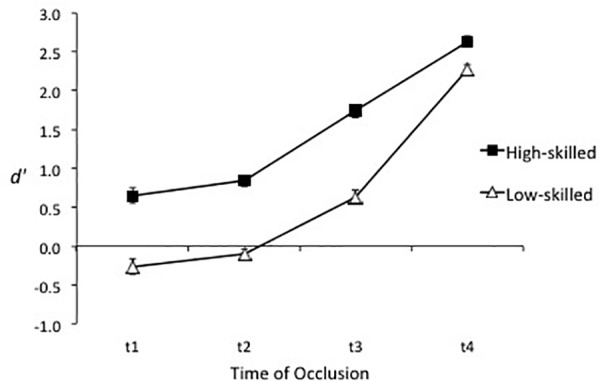
Mean sensitivity (±*SE*) for the high-skilled and low-skilled participants at each time of occlusion.

Overall discriminability was slightly higher for the full-video test (*d′* = 1.11, *SE* = 0.04) than for the point-light test (*d′* = 0.98, *SE* = 0.04); however, the difference was non-significant, *F*(1, 44) = 3.97, *p* = 0.053, $\eta_{p}^{2}$ = 0.08, as was the Test Display × Expertise interaction, *F*(1, 44) = 0.30, *p* = 0.59. Consistent with Hypothesis 1, analysis of sensitivity (*d′*) revealed that the ability to distinguish genuine and deceptive actions was substantially greater in high-skilled players (*d′* = 1.46, *SE* = 0.04) than low-skilled players (*d′* = 0.63, *SE* = 0.04), *F*(1, 44) = 175.73, *p* < 0.001, $\eta_{p}^{2}$ = 0.80. As expected given the nature of the test, the ability to distinguish between genuine and deceptive actions increased as more of the action was revealed, resulting in a significant effect of time of occlusion, *F*(1, 44) = 296.38, *p* < 0.001, $\eta_{p}^{2}$ = 0.87. The difference between high-skilled and low-skilled players was stable across t1, t2, and t3 then decreased after the foot contacted or passed in front of the ball (t4), reflected in a significant Expertise × Time of Occlusion interaction, *F*(2.4, 105.6) = 7.28, *p* = 0.001, $\eta_{p}^{2}$ = 0.14 (see Figure 3).

**FIGURE 4:**
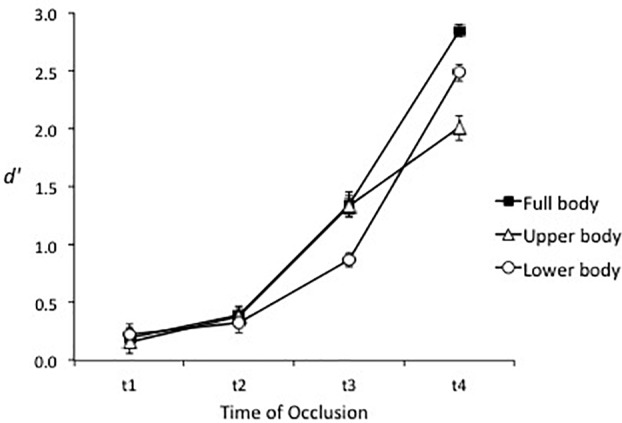
Mean sensitivity (±*SE*) in the three spatial occlusion conditions at each time of occlusion.

**FIGURE 5:**
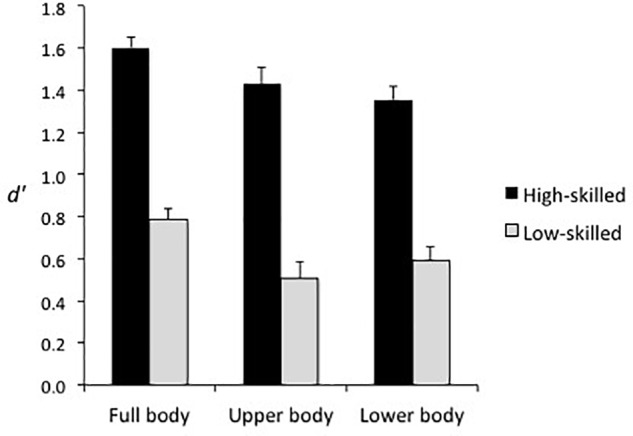
Mean sensitivity (±*SE*) for the high-skilled and low-skilled participants in the three spatial occlusion conditions.

Consistent with Hypothesis 2, sensitivity was higher in the full body condition (*d′* = 1.20, *SE* = 0.04) than the upper body condition (*d′* = 0.97, *SE* = 0.06), *F*(1, 44) = 16.26, *p* < 0.001, $\eta_{p}^{2}$ = 0.27, and lower body condition (*d′* = 0.98, *SE* = 0.04), *F*(1, 44) = 21.91, *p* < 0.001, $\eta_{p}^{2}$ = 0.33. However, a significant Spatial Occlusion × Time of Occlusion interaction, *F*(4.7, 205.7) = 11.79, *p* < 0.001, $\eta_{p}^{2}$ = 0.21, reflected that sensitivity at t3 was higher in the full body (*d′* = 1.35, *SE* = 0.11) and upper body (*d′* = 1.33, *SE* = 0.09) conditions than in the lower body condition (*d′* = 0.87, *SE* = 0.06), and that sensitivity increased more in the full body and lower body conditions than the upper body condition after the foot had been seen contacting or passing in front of the ball (t3–t4; see Figure 4). Hypothesis 3 was not supported as sensitivity for high-skilled players was greater than for low-skilled players in all spatial occlusion conditions, resulting in a non-significant Spatial Occlusion × Expertise interaction, *F*(2, 88) = 1.10, *p* = 0.34, $\eta_{p}^{2}$ = 0.02 (see Figure 5).

**FIGURE 6:**
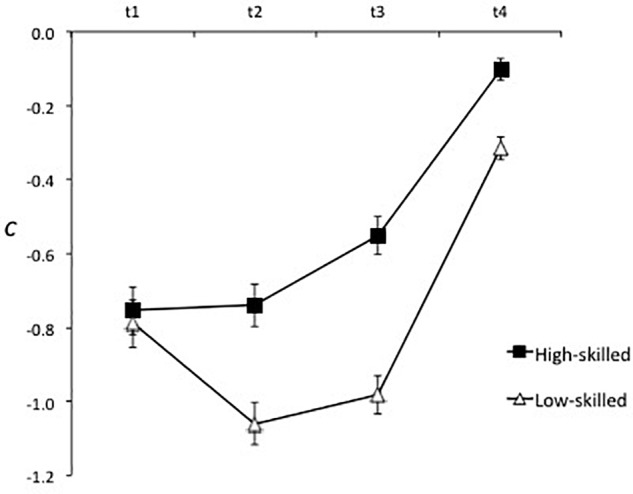
Response bias (*c*) for the high-skilled and low-skilled participants at each time of occlusion. Negative values indicate a bias toward judging the action to be genuine.

Consistent with Hypothesis 4, analysis of response bias revealed that the low-skilled players (*c* = −0.79, *SE* = 0.04) had a stronger bias toward judging actions to be genuine than the high-skilled players (*c* = −0.54, *SE* = 0.04), *F*(1, 44) = 22.86, *p* < 0.001, $\eta_{p}^{2}$ = 0.34. As can be seen in Figure 6, bias in low-skilled players was already strong at t1, increased further at t2, then stabilized at t3 before decreasing markedly at t4 after the foot had taken or passed in front of the ball. In the high-skilled players, bias was strongest at t1 and t2, decreased markedly at t3, and was almost eliminated at t4. This resulted in a significant Expertise × Time of Occlusion interaction, *F*(2.3, 103.2) = 7.87, *p* < 0.001, $\eta_{p}^{2}$ = 0.15.

**FIGURE 7:**
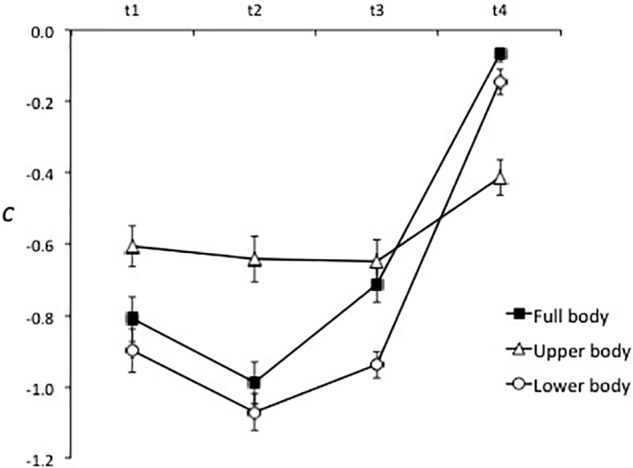
Response bias (*c*) for the three spatial occlusion conditions at each time of occlusion. Negative values indicate a bias toward judging the action to be genuine.

The hypotheses that response bias would be strongest and the expertise effect greatest in the full body condition were not supported. As can be seen in Figure 7, before veridical information became available (i.e., from t1 to t3) bias toward judging actions to be genuine was strongest in the lower body condition then full body condition, and was weakest in the upper body condition. The effect of expertise was consistent across the three conditions of spatial occlusion, resulting in a non-significant Expertise × Spatial Occlusion interaction, *F*(1.5, 66.0) = 1.35, *p* = 0.26, $\eta_{p}^{2}$ = 0.03. A significant Spatial Occlusion × Time of Occlusion interaction, *F*(4.3, 188.8) = 17.12, *p* < 0.001, $\eta_{p}^{2}$ = 0.28, reflected relatively stable bias across time of occlusion in the upper body condition in contrast to bias in the full body and lower body conditions, which strengthened from t1 to t2 then weakened at t3 and t4.

### ROC Analysis

**FIGURE 8:**
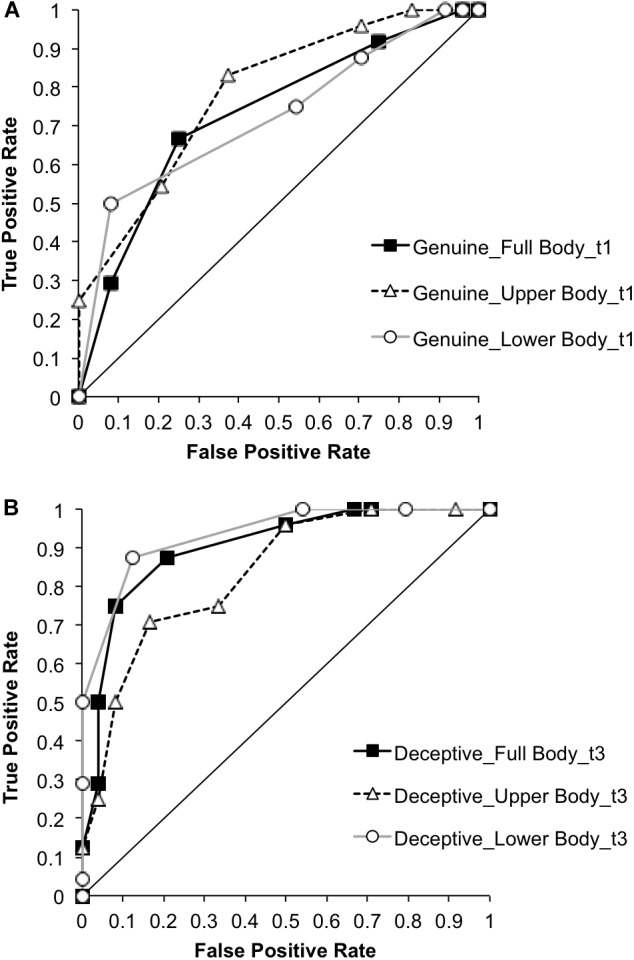
Receiver Operating Characteristic (ROC) curves for classifying participants as high-skilled or low-skilled. Panel **(A)** shows the curves for response accuracy on genuine trials occluded at t1 in each of the three spatial occlusion conditions. Panel **(B)** shows the curves for response accuracy on deceptive trials occluded at t3 in each of the three spatial occlusion conditions. If points fell along the horizontal line this would indicate an inability to classify participants into either group and would yield an area under the curve (AUC) of 0.50. Better classification of individuals as high-skilled and low-skilled is reflected by curves above and left of the horizontal line and yields AUC values greater than 0.50.

To determine the elements of the test that best categorized high-skilled and low-skilled performers, we conducted ROC analyses on the accuracy scores for genuine and deceptive trials in each spatial occlusion condition at each of the four times of occlusion. The AUC values are displayed in Table 1, in which three themes can be identified. First, most of the values are higher for trials occluded at t1, t2, and t3 than for those occluded at t4, when veridical information was available (the foot taking or passing in front of the ball). Second, the highest AUC values are found in the deceptive trials, which is consistent with the hypothesis that judgment accuracy on deceptive trials would be more “diagnostic” of expertise. Third, the highest values for genuine trials are found at t1, showing that the ability to make accurate early judgments of genuine actions distinguishes high-skilled and less-skilled performers. In contrast, the highest values for deceptive trials are found at t3, just before the foot passes in front of the ball. These findings are illustrated in Figure 8 and are characterized by the apices of ROC curves for the deceptive trials (occluded at t3; Panel B) bowing further from the diagonal line than those for genuine trials (occluded at t1; Panel A). In contrast to the hypothesis that the highest AUC values would be in the full body deceptive trials, judgment accuracy for deceptive trials in the lower body condition (AUC = 0.94) and full body condition (AUC = 0.90) distinguished expertise extremely well and slightly better than deceptive trials in the upper body condition (AUC = 0.83; Figure 8B).

**Table 1:** Area under the curve (AUC) values from the receiver operating characteristic analysis.

		t1			t2			t3			t4		
		AUC	*p*	95% CI	AUC	*p*	95% CI	AUC	*p*	95% CI	AUC	*p*	95% CI
Genuine	Full body	**0.73**	0.01	0.59–0.87	0.51	0.90	0.35–0.68	0.62	0.16	0.46–0.78	0.46	0.61	0.29–0.62
	Upper body	**0.79**	0.00	0.66–0.91	0.56	0.49	0.39–0.72	0.63	0.13	0.47–0.79	0.52	0.84	0.35–0.68
	Lower body	**0.72**	0.01	0.58–0.87	0.67	0.04	0.52–0.83	0.66	0.06	0.50–0.82	0.51	0.93	0.34–0.67
Deceptive	Full body	0.68	0.03	0.53–0.84	0.87	0.00	0.78–0.97	**0.90**	0.00	0.81–0.99	0.76	0.00	0.62–0.90
	Upper body	0.76	0.00	0.62–0.89	0.82	0.00	0.70–0.94	**0.83**	0.00	0.72–0.95	0.74	0.01	0.60–0.88
	Lower body	0.67	0.05	0.51–0.82	0.84	0.00	0.72–0.95	**0.94**	0.00	0.87–1.00	0.72	0.01	0.57–0.87

*The values indicate how well different elements of the test differentiate between the binary classifier (high-skilled and low-skilled). P-values indicate AUC values that are significantly greater than 0.5, which represents classification at chance level. 95% confidence intervals (CI) associated with the AUC values are also stated. Values in bold are the highest associated with the genuine and deceptive trials and ROC curves for these are presented in Figure 8.*

## Discussion

Expertise in perceiving deceptive intent has been linked to an ability to attend to ‘honest’ signals, such as center of mass, while ignoring deceptive signals (Brault et al., 2012). This fits with the narrative that high-skilled performers use ‘global’ information from distributed sources whereas less-skilled performers are more reliant on local sources of (potentially deceptive) information (Ward et al., 2002). If true, skilled perception of deceptive intent may involve processing the same sources of visual information in a different, more holistic manner, rather than enhanced sensitivity to the critical sources that convey deceptive intent. This would have significant implications for perceptual training protocols, for example, in regard to the degree to which performers should be made aware of information linked to local sources as opposed to more global, relational information. To test this experimentally we manipulated the sources of information available to participants as they attempted to judge the direction a football player would take the ball by determining whether the initial intention conveyed by the player was genuine or fake. Overall, we found clear differences between the performance of high-skilled and low-skilled performers that were consistent across the full-video and point-light tests, highlighting the importance of kinematic information in anticipation and judgment of deceptive intent (Abernethy et al., 2001, 2008).

The results of the signal detection analysis revealed that high-skilled participants were better at differentiating genuine and deceptive actions and were most sensitive on trials occluded before the foot contacted or passed in front of the ball (t3). Averaged across all spatial occlusion conditions they made proportionately more correct responses to genuine actions (‘hits’) than they made incorrect responses to deceptive actions (‘false alarms’), which yielded positive values of *d′*. This contrasts with the overall performance of low-skilled participants, who made fewer correct responses to genuine actions than incorrect responses to deceptive actions on trials occluded at t1 and t2, resulting in negative values of *d′*. Negative sensitivity values are uncommon in most of the tasks in which signal detection analysis is used; however, they can be accounted for by use of exaggerated movements to convey a false intention (Brault et al., 2010). Exaggeration has been shown to make some actions more recognizable (Pollick et al., 2001) so when exaggeration is associated with deceptive actions it is logical that the proportion of false alarms can exceed the proportion of hits. Negative sensitivity scores (higher proportions of false alarms than hits) were also found in groups of police investigators and trained students who judged the innocence or guilt of individuals in mock crime interviews (Meissner and Kassin, 2002). Collectively, these scores reveal a key attribute of skilled judgments of deceptive intent, namely the ability to differentiate genuine and deceptive actions earlier in the action sequence. Analysis of the ROC curves confirms it is the ability to judge deceptive actions that best differentiates the two groups (Jackson and Cañal-Bruland, in press; Wright and Jackson, 2014).

The analysis also revealed a clear pattern of results with respect to response bias. There was support for the hypothesis that low-skilled performers would show a stronger bias toward judging actions to be genuine than would high-skilled performers. Moreover, the strength of this bias in the low-skilled group increased from t1 to t2, and in both groups weakened considerably after the player’s foot contacted or passed in front of the ball. Combined with the instructions participants received regarding the equal number of genuine and deceptive trials, this implies that the main source of bias was perceptual, which reflects the goal of the actor in conveying a false intention. Low-skilled participants were fooled more frequently so made more ‘false alarm’ responses, peaking at trials occluded at t2 and decreasing considerably after veridical information became available. High-skilled players were fooled less frequently and bias peaked earlier in the action sequence (t1), which supports the interpretation of earlier differentiation of genuine and deceptive actions.

In regard to the sources of information that support accurate judgments of deceptive intent, high-skilled players had the same advantage in sensitivity over low-skilled participants in the full body, lower body, and upper body occlusion conditions (see Figure 5). This indicates that information from the lower body or upper body was sufficient to support the expertise effect but that global information, or other relational information concurrently derived from both sources, was not necessary. Instead, the data suggest that high-skilled players were more sensitive than low-skilled players to kinematic information from both the lower and upper body. The picture is a little more nuanced in that upper body and lower body information appear to have been processed sequentially or weighted differently across times of occlusion (Figure 4). Specifically, in all three spatial occlusion conditions sensitivity was very low at t1 and improved very little from t1 to t2. Sensitivity then increased more from t2 to t3 when the upper body was visible but improved more from t3 to t4 when the lower body was visible. Information from the upper body was therefore more useful for early differentiation of genuine and deceptive actions while veridical information provided by the lower body became dominant later in the action. Consistent with this interpretation, the effect of spatial occlusion on response bias was stronger for the full body and lower body conditions than for the upper body condition in early-occluded trials, which implies that the lower body was the primary source for conveying deception. This was supported by attenuation of bias in the full body and lower body conditions after the foot contacted or passed in front of the ball (Figure 7).

Relating our findings to those of Brault et al. (2012), it is important to note that while tau of COM displacement (the ratio between current motion-gap size and its rate of closure) accounted for most of the variance (74%) in expert responses to rugby sidesteps the deceptive signals accounted for more than 50% of the variance. Some signals (e.g., head yaw) have minimal impact upon COM displacement, which suggests that expert sensitivity extended beyond a globally derived source to assessing the veracity of more local deceptive sources. Williams et al. (2009) argued that by using distributed sources of information high-skilled players might be harder to deceive because they would be more resistant to local perturbations. Our results are consistent with this insofar as there was no advantage for globally derived information over information gleaned from local sources, at least in the coarse distinction between lower body and upper body sources. The results are also consistent with research showing that kinematic differences between non-deceptive and deceptive actions span multiple markers across the upper and lower body (Smeeton and Williams, 2012). At the same time, while our results suggest sequential processing of local information from the upper and lower body they do not preclude holistic processing of information within each source. Indeed, Lopes et al. (2014) found that the variables that best differentiated genuine and deceptive football penalty kicks were stronger predictors of kick direction when expressed as a compound variable. By implication, deceptive actions may be most effective when the player attends to specific isolated cues within a broader source. In the present task attending to the lead foot as it gathered or passed in front of the ball ultimately provided veridical information about the player’s intentions but was also the primary source for conveying a false intention. Sequential attention to different sources of information in discrete tasks was shown in a study of expert futsal goalkeepers while they faced penalty kicks (Navia et al., 2017). The goalkeepers focused predominantly on the penalty taker’s head during the early phase of the run up then on the ball in the final stride of the player’s approach. A similar analysis of the spatiotemporal characteristics of visual gaze in judgments of stepover actions may provide corroborative evidence for sequential processing of lower and upper body information.

## Conclusion

It is becoming increasingly clear that high-skilled performers have a sizeable advantage over less-skilled performers in their ability to judge deceptive intent. The present study shows how signal detection analyses can be used to capture the essence of these tasks, which is to discriminate between a genuine and deceptive action. This analysis revealed that the advantage of high-skilled football players resides in their ability to use information from both the lower and upper body, yet also showed that they are not dependent on global information concurrently derived from these sources. Moreover, expertise was reflected in different levels of (perceptual) bias toward judging actions to be genuine. Last, ROC analysis revealed that, within the context of a task that contains both genuine and deceptive actions, judgment accuracy for deceptive actions strongly differentiates high-skilled and low-skilled performers. How information from different sources is used to resolve genuine and deceptive actions, and the extent to which the present results relate to *in situ* physical responses, warrants further investigation. Some researchers have shown no discrepancy between verbal and physical responses (Jackson and Mogan, 2007) while others have found that expertise effects are greater when participants make coupled physical responses (Mann et al., 2010). In addition, other sources of bias warrant further investigation. In sport, performers commonly have knowledge of situational probabilities regarding player preferences. We expect that this will bias performer responses and there are early indications that this is the case (Jackson and Barton, 2018). How such information and other sources of bias affect response sensitivity is critical for developing a full understanding of how anticipation skill relates to judgments of deceptive intent.

## Ethics Statement

This study was carried out in accordance with the recommendations of Brunel University London Institutional Ethics Committee with written informed consent from all subjects. All subjects gave written informed consent in accordance with the Declaration of Helsinki. The protocol was approved by the Brunel University London Institutional Ethics Committee.

## Author Contributions

RJ led design of the study, analysis, and writing of the paper. HB contributed to the design of the study and drafting of the paper. KA and BA contributed to the design of the study and writing of the paper.

## Conflict of Interest Statement

The authors declare that the research was conducted in the absence of any commercial or financial relationships that could be construed as a potential conflict of interest.

## References

[B1] Abernethy B. Gill D. P. Parks S. L. Packer S. T. (2001). Expertise and the perception of kinematic and situational probability information. *Perception* 30 233–252. 10.1068/p2872 11296504

[B2] Abernethy B. Russell D. G. (1987). The relationship between expertise and visual search strategy in a racquet sport. *Hum. Mov. Sci.* 6 283–319. 10.1016/0167-9457(87)90001-7

[B3] Abernethy B. Zawi K. Jackson R. C. (2008). Expertise and attunement to kinematic constraints. *Perception* 37 931–948. 10.1068/p5340 18686711

[B4] Biscombe T. Drewitt P. (1997). *Rugby: Steps to Success.* London: Human Kinetics.

[B5] Bishop D. T. Wright M. J. Jackson R. C. Abernethy B. (2013). Neural bases for anticipation skill in soccer: an fMRI study. *J. Sport Exerc. Psychol.* 35 98–109. 10.1123/jsep.35.1.98 23404883

[B6] Brault S. Bideau B. Craig C. Kulpa R. (2010). Balancing deceit and disguise: how to successfully fool the defender in a 1 vs. 1 situation in rugby. *Hum. Mov. Sci.* 29 412–425. 10.1016/j.humov.2009.12.004 20417980

[B7] Brault S. Bideau B. Kulpa R. Craig C. M. (2012). Detecting deception in movement: the case of the side-step in rugby. *PLoS One* 7:e37494. 10.1371/journal.pone.0037494 22701569 PMC3372470

[B8] Cañal-Bruland R. Schmidt M. (2009). Response bias in judging deceptive movements. *Acta Psychol.* 130 235–240. 10.1016/j.actpsy.2008.12.009 19193359

[B9] Cañal-Bruland R. van Ginneken W. F. van der Meer B. R. Williams A. M. (2011). The effect of local kinematic changes on anticipation judgments. *Hum. Mov. Sci.* 30 495–503. 10.1016/j.humov.2010.10.001 21239078

[B10] Diaz G. J. Fajen B. R. Phillips F. (2012). Anticipation from biological motion: the goalkeeper problem. *J. Exp. Psychol. Hum. Percept. Perform.* 38 848–864. 10.1037/a0026962 22309088

[B11] Faul F. Erdfelder E. Lang A.-G. Buchner A. (2007). G^∗^Power 3: a flexible statistical power analysis program for the social, behavioral, and biomedical sciences. *Behav. Res. Methods* 39 175–191. 10.3758/BF03193146 17695343

[B12] Green D. M. Swets J. A. (1966). *Signal Detection Theory and Psychophysics.* London: Wiley.

[B13] Güldenpenning I. Steinke A. Koester D. Schack T. (2013). Athletes and novices are differently capable to recognize feint and non-feint actions. *Exp. Brain Res.* 230 333–343. 10.1007/s00221-013-3658-2 23955103

[B14] Huys R. Cañal-Bruland R. Hagemann N. Beek P. J. Smeeton N. J. Williams A. M. (2009). Global information pickup underpins anticipation of tennis shot direction. *J. Mot. Behav.* 41 158–170. 10.3200/JMBR.41.2.158-171 19201686

[B15] Huys R. Smeeton N. J. Hodges N. J. Beek P. J. Williams A. M. (2008). On the dynamic information underlying visual anticipation skill. *Percept. Psychophys.* 70 1217–1234. 10.3758/PP.70.7.1217 18927005

[B16] Jackson R. C. Barton H. (2018). “Actions become ‘super-deceptive’ when preceded by (congruent) situational probability information,” in *Proceedings of the North American Society for the Psychology of Sport and Physical Activity Annual Conference*, Denver, CO.

[B17] Jackson R. C. Cañal-Bruland R. (in press). “Deception in sport,” in *Anticipation and Decision Making in Sport*, eds Williams A. M. Jackson R. C. (Abingdon: Routledge).

[B18] Jackson R. C. Mogan P. (2007). Advance visual information, awareness, and anticipation skill. *J. Mot. Behav.* 39 341–351. 10.3200/JMBR.39.5.341-352 17827112

[B19] Jackson R. C. Warren S. Abernethy B. (2006). Anticipation skill and susceptibility to deceptive movement. *Acta Psychol.* 123 355–371. 10.1016/j.actpsy.2006.02.002 16546104

[B20] Johansson G. (1973). Visual perception of biological motion and a model for its analysis. *Percept. Psychophys.* 14 201–211. 10.3758/BF03212378

[B21] Loffing F. Hagemann N. (2014). Skill differences in visual anticipation of type of throw in team-handball penalties. *Psychol. Sport Exerc.* 15 260–267. 10.1016/j.psychsport.2014.01.006

[B22] Lopes J. E. Jacobs D. M. Travieso D. Araujo D. (2014). Predicting the lateral direction of deceptive and non-deceptive penalty kicks in football from the kinematics of the kicker. *Hum. Mov. Sci.* 36 199–216. 10.1016/j.humov.2014.04.004 24846289

[B23] Mann D. L. Abernethy B. Farrow D. (2010). Action specificity increases anticipatory performance and the expert advantage in natural interceptive tasks. *Acta Psychol.* 135 17–23. 10.1016/j.actpsy.2010.04.006 20507831

[B24] Mann D. T. Y. Williams A. M. Ward P. Janelle C. M. (2007). Perceptual-cognitive expertise in sport: a meta-analysis. *J. Sport Exerc. Psychol.* 29 457–478. 10.1123/jsep.29.4.457 17968048

[B25] Meissner C. A. Kassin S. M. (2002). “He’s guilty!”: investigator bias in judgments of truth and deception. *Law Hum. Behav.* 26 469–480. 10.1023/A:1020278620751 12412493

[B26] Mori S. Shimada T. (2013). Expert anticipation from deceptive action. *Atten. Percept. Psychophys.* 75 751–770. 10.3758/s13414-013-0435-z 23436250

[B27] Müller S. Abernethy B. Eid M. McBean R. Roses M. (2010). Expertise and the spatio-temporal characteristics of anticipatory information pick-up from complex movement patterns. *Perception* 39 745–760. 10.1068/p6438 20698470

[B28] Müller S. Abernethy B. Farrow D. (2006). How do world-class cricket batsmen anticipate a bowler’s intention? *Q. J. Exp. Psychol.* 59 2162–2186. 10.1080/02643290600576595 17095494

[B29] Navia J. A. Dicks M. van der Kamp J. Ruiz L. M. (2017). Gaze control during interceptive actions with different spatiotemporal demands. *J. Exp. Psychol. Hum. Percept. Perform.* 43 783–793. 10.1037/xhp0000347 28345945

[B30] Pollick F. E. Fidopiastis C. Braden V. (2001). Recognising the style of spatially exaggerated tennis serves. *Perception* 30 323–338. 10.1068/p3064 11374203

[B31] Ripoll H. Kerlirzin Y. Stein J.-F. Reine B. (1995). Analysis of information processing, decision making, and visual strategies in complex problem solving sport situations. *Hum. Mov. Sci.* 14 325–349. 10.1016/0167-9457(95)00019-O

[B32] Sebanz N. Shiffrar M. (2009). Detecting deception in a bluffing body: the role of expertise. *Psychon. Bull. Rev.* 16 170–175. 10.3758/PBR.16.1.170 19145029

[B33] Simpson P. Hesse U. (2013). *Who invented the stepover?.* London: Profile Books.

[B34] Smeeton N. J. Williams A. M. (2012). The role of movement exaggeration in the anticipation of deceptive soccer penalty kicks. *Br. J. Psychol.* 103 539–555. 10.1111/j.2044-8295.2011.02092.x 23034111

[B35] Stanislaw H. Todorov N. (1999). Calculation of signal detection theory measures. *Behav. Res. Methods Instrum. Comput.* 31 137–149. 10.3758/BF03207704 10495845

[B36] Swets J. A. (2014). *Signal Detection Theory and ROC Analysis in Psychology and Diagnostics: Collected Papers.* London: Taylor & Francis Group.

[B37] Vicente K. J. Wang J. H. (1998). An ecological theory of expertise effects in memory recall. *Psychol. Rev.* 105 33–57. 10.1037/0033-295X.105.1.33 9450371

[B38] Ward P. Williams A. M. Bennett S. J. (2002). Visual search and biological motion perception in tennis. *Res. Q. Exerc. Sport* 73 107–112. 10.1080/02701367.2002.10608997 11926480

[B39] Williams A. M. Huys R. Cañal-Bruland R. Hagemann N. (2009). The dynamical information underpinning anticipation skill. *Hum. Mov. Sci.* 28 362–370. 10.1016/j.humov.2008.10.006 19081648

[B40] Witt J. K. Taylor J. E. T. Sugovic M. Wixted J. T. (2015). Signal detection measures cannot distinguish perceptual biases from response biases. *Perception* 44 289–300. 10.1068/p7908 26562253

[B41] Wixted J. T. Mickes L. (2014). A signal-detection-based diagnostic-feature-detection model of eyewitness identification. *Psychol. Rev.* 121 262–276. 10.1037/a0035940 24730600

[B42] Wright M. J. Bishop D. T. Jackson R. C. Abernethy B. (2013). Brain regions concerned with the identification of deceptive soccer moves by higher-skilled and lower-skilled players. *Front. Hum. Neurosci.* 7:851. 10.3389/fnhum.2013.00851 24381549 PMC3865769

[B43] Wright M. J. Jackson R. C. (2014). Deceptive body movements reverse spatial cueing in Soccer. *PLoS One* 9:e104290. 10.1371/journal.pone.0104290 25100444 PMC4123942

[B44] Zweig M. H. Campbell G. (1993). Receiver-operating characteristic (ROC) plots: a fundamental evaluation tool in clinical medicine. *Clin. Chem.* 39 561–577. 8472349

